# A guide to selecting high-performing antibodies for DJ-1 ( *PARK7*) (Q99497) for use in western blot, immunoprecipitation, and immunofluorescence

**DOI:** 10.12688/f1000research.182633.1

**Published:** 2026-06-23

**Authors:** CongYao Zha, Charles Alende, Maryam Fotouhi, Sara González Bolívar, Riham Ayoubi, Vincent Francis, Peter S. McPherson, Carl Laflamme

**Affiliations:** 1 Department of Neurology and Neurosurgery, Structural Genomics Consortium, The Montreal Neurological Institute, McGill University, Montreal, Canada

**Keywords:** UniProt ID Q99497, PARK7, DJ-1, Parkinson disease protein 7, antibody characterization, antibody validation, western blot, immunoprecipitation, immunofluorescence

## Abstract

DJ-1 is a multifunctional protein that plays a pivotal role in cellular protection against oxidative stress and neurodegeneration. Mutations in the *PARK7* gene are associated with early-onset familial Parkinson’s disease. Here we have characterized sixteen DJ-1 commercial antibodies for western blot, immunoprecipitation, and immunofluorescence using a standardized experimental protocol based on comparing read-outs in knockout cell lines and isogenic parental controls. These studies are part of a larger, collaborative initiative seeking to address antibody reproducibility issues by characterizing commercially available antibodies for human proteins and publishing the results openly as a resource for the scientific community. While the use of antibodies and protocols vary between laboratories, we encourage readers to use this report as a guide to select the most appropriate antibodies for their specific needs.

## Introduction

DJ-1 is a highly conserved, redox-sensitive protein that plays a role in maintaining cellular homeostasis, regulating oxidative stress and preserving mitochondrial integrity. ^1^ In neurons, DJ-1 stabilizes mitochondria by reducing oxidative stress and promoting the clearance of dysfunctional mitochondria through mitophagy, thereby preventing the accumulation of toxic reactive oxygen species (ROS) and mitigating neuronal damage. ^2^ Beyond its protective role against oxidative damage, DJ-1 has been implicated in diverse cellular processes, including regulation of transcription factors, protection from protein aggregation, modulation of apoptosis, and maintenance of mitochondrial dynamics. Mutations in the *PARK7* gene, which cause loss of function, are linked to early-onset familial Parkinson’s disease (PD) by predisposing dopaminergic neurons to degeneration. ^3^

This research is part of a broader collaborative initiative in which academics, funders and commercial antibody manufacturers are working together to address antibody reproducibility issues by characterizing commercial antibodies for human proteins using standardized protocols, and openly sharing the data. ^4^ It consists of identifying human cell lines with adequate target protein expression and the development/contribution of equivalent knockout (KO) cell lines, followed by antibody characterization procedures using most commercially available renewable antibodies against the corresponding protein. ^4^ Here we characterized sixteen commercial DJ-1 antibodies, selected and donated by participant antibody manufacturers, for use in western blot, immunoprecipitation, and immunofluorescence (also referred to as immunocytochemistry), enabling biochemical and cellular assessment of DJ-1 properties and function.

The authors do not engage in result analysis or offer explicit antibody recommendations. Our primary aim is to deliver top-tier data to the scientific community, grounded in Open Science principles. This empowers experts to interpret the characterization data independently, enabling them to make informed choices regarding the most suitable antibodies for their specific experimental needs. Guidelines on how to interpret antibody characterization data found in this study are featured on the YCharOS gateway ^5^ and in Table 4 of this data note. ^4^

## Results and discussion

Our standard protocol involves comparing readouts from wild type (WT) and KO cells. ^6, 7^ The first step is to identify a cell line that expresses the target protein at sufficient levels to generate a measurable signal. Using the DepMap transcriptomics database (Cancer Dependency Map Portal, RRID: SCR_017655), we found that DJ-1 is highly and ubiquitously expressed across cancer cell lines. Accordingly, a *PARK7* KO HEK-293 T cell line was obtained from Abcam ( Table 1).

**Table 1.** Summary of the cell lines used.

Institution	Catalog number	RRID (Cellosaurus)	Cell line	Genotype
Abcam	ab255449	CVCL_0063	HEK-293 T	WT
Abcam	ab266338	CVCL_B3DD	HEK-293 T	*PARK7* KO

**Figure 1.**
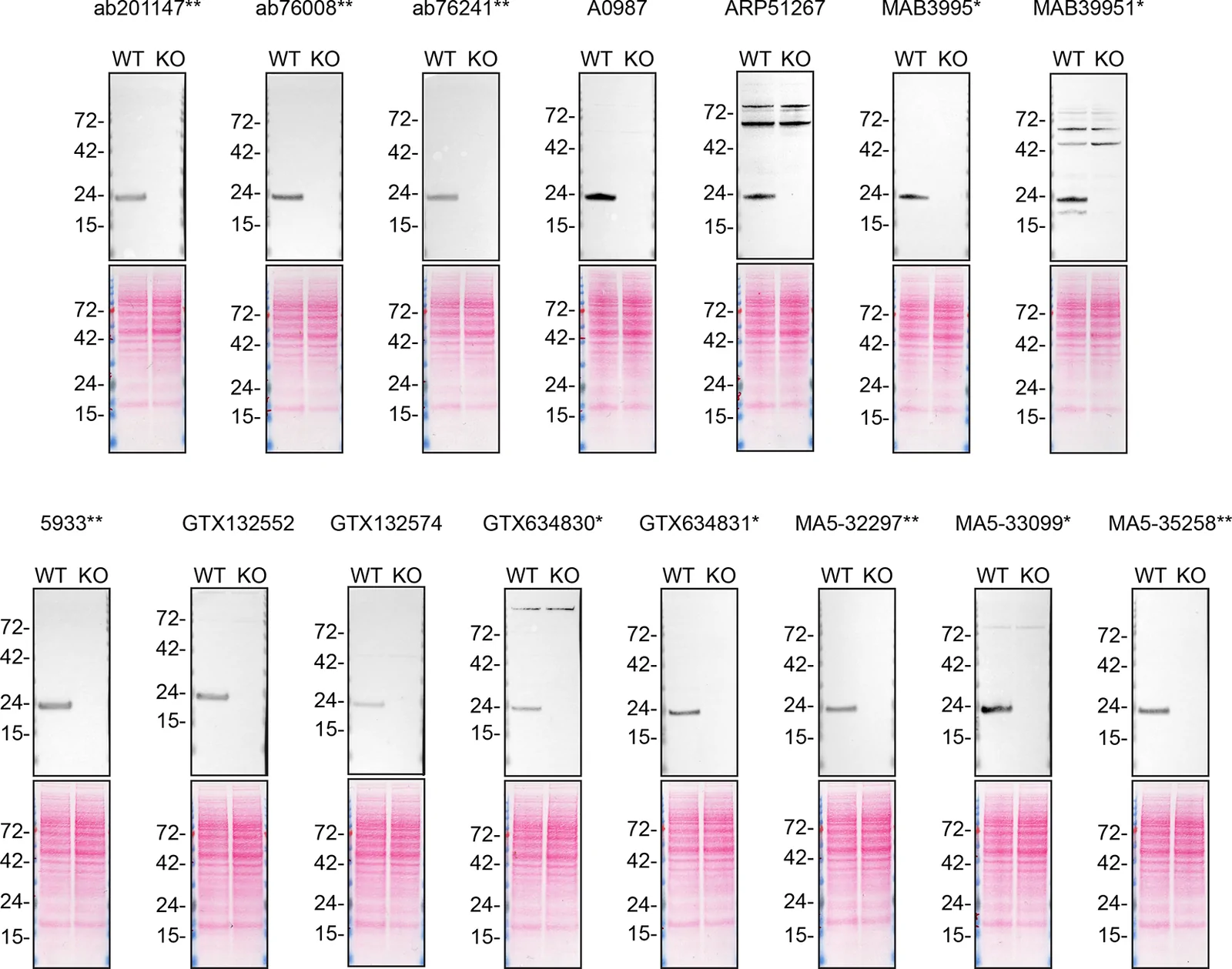
DJ-1 antibody screening by western blot. Lysates of HEK-293 T WT and *PARK7* KO were prepared, and 30 μg of protein were processed for western blot with the indicated DJ-1 antibodies. The Ponceau stained transfers of each blot are presented to show equal loading of WT and KO lysates and protein transfer efficiency from the acrylamide gels to the nitrocellulose membrane. Antibody dilutions were chosen according to the recommendations of the antibody supplier. Antibody dilutions used: ab201147** at 1/5000; ab76008** at 1/5000; ab76241** at 1/2000; A0987 at 1/1000; ARP51267 at 1/500; MAB3995* at 1/1000; MAB39951* at 1/500; 5933** at 1/1000; GTX132552 at 1/2000; GTX132574 at 1/2000; GTX634830* at 1/2000; GTX634831* at 1/5000; MA5–32297** at 1/1000; MA5–33099* at 1/2000; MA5–35258** at 1/1000. Predicted band size: 20 kDa. * = monoclonal antibody, ** = recombinant antibody.

To screen all sixteen antibodies by western blot, WT and *PARK7* KO protein lysates were ran on SDS-PAGE, transferred onto nitrocellulose membranes, and then probed with sixteen DJ-1 antibodies in parallel ( Figure 1).

**Figure 2.**
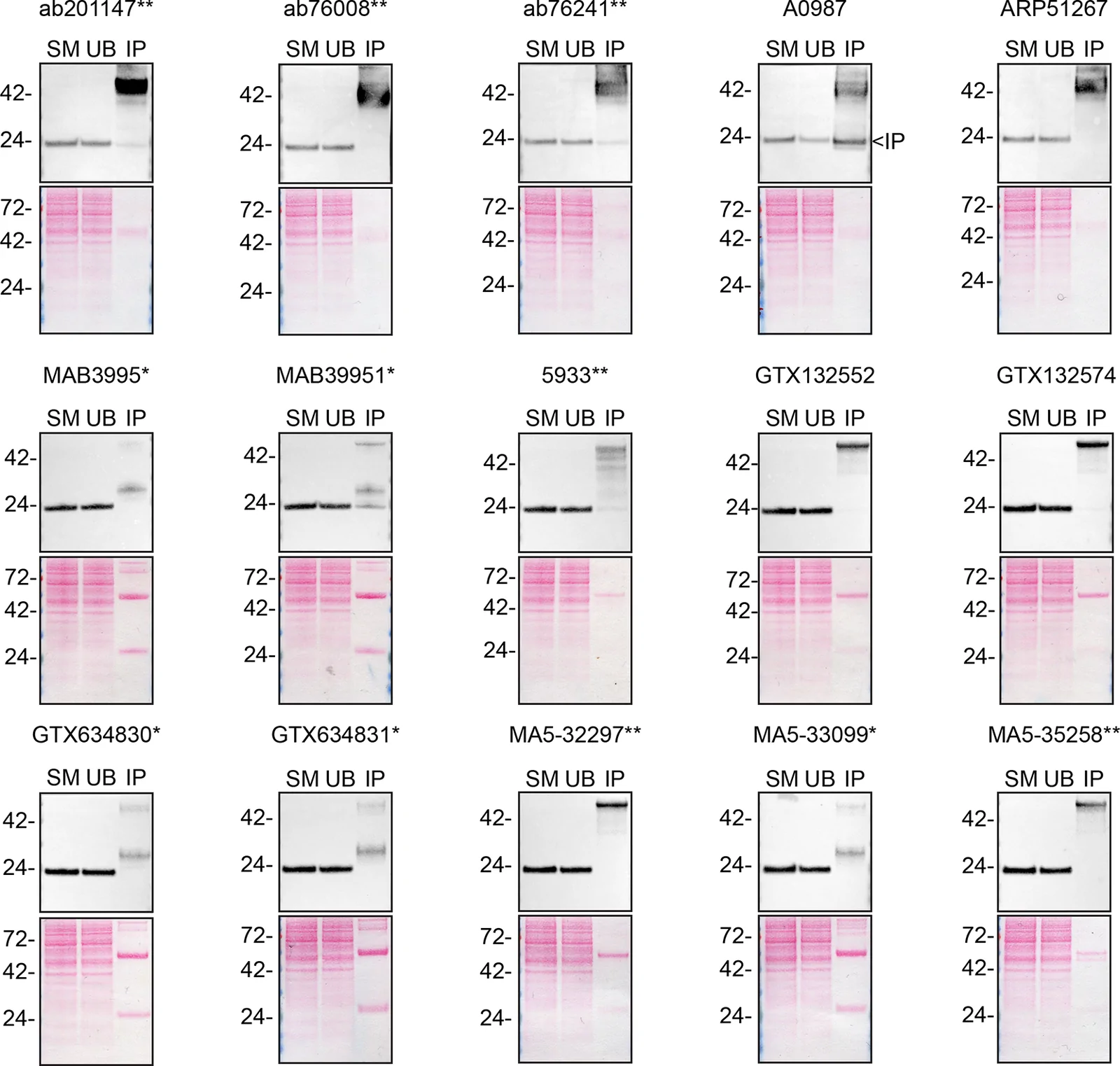
DJ-1 antibody screening by immunoprecipitation. HEK-293 T lysates were prepared, and immunoprecipitation was performed using 0.5 mg of lysate and 2.0 μg of the indicated DJ-1 antibodies pre-coupled to Dynabeads protein A or protein G. Samples were washed and processed for western blot with the DJ-1 antibody ab76008** used at 1/5000. The Ponceau stained transfers of each blot are shown. SM = 6% starting material; UB = 6% unbound fraction; IP = immunoprecipitate. * = monoclonal antibody, ** = recombinant antibody.

We then assessed the capability of all sixteen antibodies to capture DJ-1 from HEK-293 T protein extracts using immunoprecipitation techniques, followed by western blot analysis. For the immunoblot step, a specific DJ-1 antibody identified previously (refer to Figure 1) was selected. Equal amounts of the starting material (SM) and the unbound fractions (UB), as well as the whole immunoprecipitate (IP) eluates were separated by SDS-PAGE ( Figure 2).

**Figure 3.**
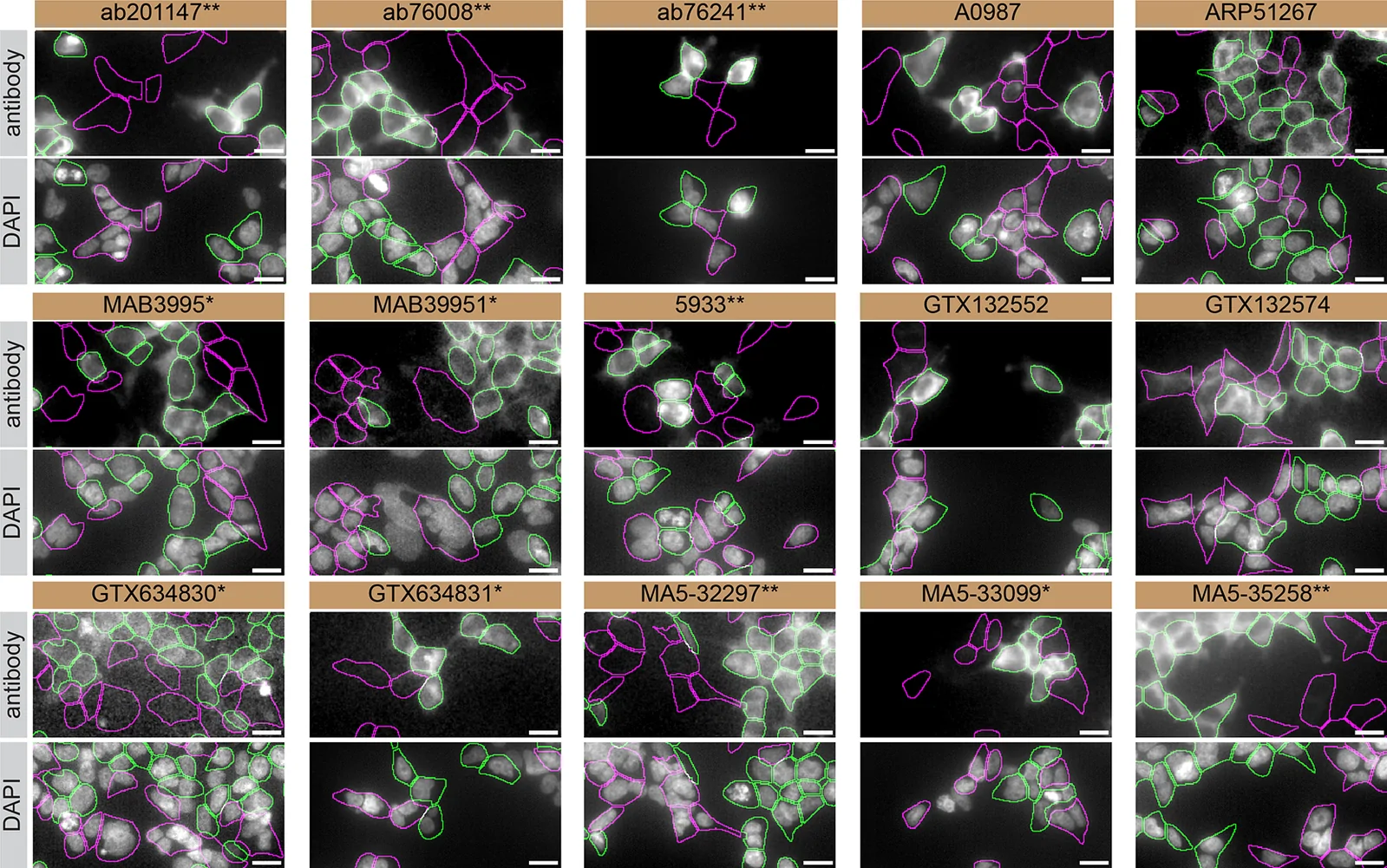
DJ-1 antibody screening by immunofluorescence. HEK-293 T WT and *PARK7* KO cells were labelled with a green or a far-red fluorescent dye, respectively. WT and KO cells were mixed and plated to a 1:1 ratio on a 96-well collagen-coated plate with optically clear flat-bottom. Cells were stained with the indicated DJ-1 antibodies and with the corresponding Alexa-fluor 555 coupled secondary antibody including DAPI. Acquisition of the blue (nucleus-DAPI), green (WT), red (antibody staining) and far-red (KO) channels was performed. Representative images of the blue and red (grayscale) channels are shown. WT and KO cells are outlined with green and magenta dashed line, respectively. When an antibody was recommended for immunofluorescence by the supplier, we tested it at the recommended dilution. The rest of the antibodies were tested at 1 and 2 μg/ml, and the final concentration was selected based on the detection range of the microscope used and a quantitative analysis not shown here. Antibody dilutions used: ab201147** at 1/500; ab76008** at 1/100; ab76241** at 1/100; A0987 at 1/1500; ARP51267 at 1/200; MAB3995* at 1/500; MAB39951* at 1/500; 5933** at 1/50; GTX132552 at 1/100; GTX132574 at 1/500; GTX634830* at 1/1400; GTX634831* at 1/500; MA5–32297** at 1/1000; MA5–33099* at 1/100; MA5–35258** at 1/60. Bars = 10 μm. * = monoclonal antibody, ** = recombinant antibody.

For immunofluorescence, the sixteen DJ-1 antibodies were screened using a mosaic strategy. First, HEK-293 T WT and *PARK7* KO cells were labelled with different fluorescent dyes in order to distinguish the two cell lines, and the DJ-1 antibodies were evaluated. Both WT and KO lines imaged in the same field of view to reduce staining, imaging and image analysis bias ( Figure 3). Quantification of immunofluorescence intensity in hundreds of WT and KO cells was performed for each antibody tested, and the images presented in Figure 3 are representative of this analysis. ^4^

In conclusion, we have screened sixteen DJ-1 commercial antibodies by western blot, immunoprecipitation, and immunofluorescence by comparing the signal produced by the antibodies in human HEK-293 T WT and *PARK7* KO cells. To assist users in interpreting antibody performanyce, Table 4 outlines various scenarios in which antibodies may perform in all three applications. ^8^ High-quality and renewable antibodies that successfully detect DJ-1 antibodies were identified. Researchers who wish to study DJ-1 in a different species are encouraged to select high-quality antibodies, based on the results of this study, and investigate the predicted species reactivity of the manufacturer before extending their research.

## Limitations

Inherent limitations are associated with the antibody characterization platform used in this study. Firstly, the YCharOS project focuses on renewable (recombinant and monoclonal) antibodies and does not test all commercially available DJ-1 antibodies. YCharOS partners provide approximately 80% of all renewable antibodies, but some top-cited polyclonal antibodies may not be available through these partners. We encourage readers to consult vendor documentation to identify the specific antigen each antibody is raised against, where such information is available.

Secondly, the YCharOS effort employs a non-biased approach that is agnostic to the protein for which antibodies have been characterized. The aim is to provide objective data on antibody performance without preconceived notions about how antibodies should perform or the molecular weight that should be observed in western blot. As the authors are not experts in DJ-1, only a brief overview of the protein’s function and its relevance in disease is provided. DJ-1 experts are invited to analyze and interpret observed banding patterns in western blots and subcellular localization in immunofluorescence.

Thirdly, YCharOS experiments are not performed in replicates primarily due to the use of multiple antibodies targeting various epitopes. Once a specific antibody is identified, it validates the protein expression of the intended target in the selected cell line, confirms the lack of protein expression in the KO cell line and supports conclusions regarding the specificity of the other antibodies. All experiments are performed using master mixes, and meticulous attention is paid to sample preparation and experimental execution. In IF, the use of two different concentrations serves to evaluate antibody specificity and can aid in assessing assay reliability. In instances where antibodies yield no signal, a repeat experiment is conducted following titration. Additionally, our independent data is performed subsequently to the antibody manufacturers internal validation process, therefore making our characterization process a repeat.

Lastly, as comprehensive and standardized procedures are respected, any conclusions remain confined to the experimental conditions and cell line used for this study. The use of a single cell type for evaluating antibody performance poses as a limitation, as factors such as target protein abundance significantly impact results. Additionally, the use of cancer cell lines containing gene mutations poses a potential challenge, as these mutations may be within the epitope coding sequence or other regions of the gene responsible for the intended target. Such alterations can impact the binding affinity of antibodies. This represents an inherent limitation of any approach that employs cancer cell lines.

## Method

The standardized protocols used to carry out this KO cell line-based antibody characterization platform was established and approved by a collaborative group of academics, industry researchers and antibody manufacturers. The detailed materials and step-by-step protocols used to characterize antibodies in western blot, immunoprecipitation and immunofluorescence are openly available on Protocols.io ( protocols.io/view/a-consensus-platform-for-antibody-characterization ). ^4^ Brief descriptions of the experimental setup used to carry out this study can be found below.

### Cell lines and antibodies

The cell lines, primary and secondary antibodies used in this study are listed in Table 1, 2, and 3, respectively. To ensure consistency with manufacturer recommendations and account for proprietary formulations (where antibody concentrations are not disclosed), antibody usage is reported as dilution ratios rather than absolute concentrations. To facilitate proper citation and unambiguous identification, all cell lines and antibodies are referenced with their corresponding Research Resource Identifiers (RRIDs). ^9, 10^ All cell lines used in this study were regularly tested for mycoplasma contamination and were confirmed to be mycoplasma-free.

**Table 2.** Summary of the DJ-1 antibodies tested.

Company	Catalog number	Lot number	RRID (Antibody Registry)	Clonality	Clone ID	Host	Concentration (μg/μL)	Vendors recommended applications
Abcam	ab201147 **	1106441–1	AB_1310549	recombinant mono	EPR19466–105	rabbit	0.52	Wb, IP, IF
Abcam	ab76008 **	1018054–21	AB_2283514	recombinant mono	EP2815Y	rabbit	0.11	Wb, IP, IF
Abcam	ab76241 **	1097332–2	AB_2757506	recombinant mono	EP2816Y	rabbit	0.26	Wb
ABclonal	A0987	0001720101	AB_2047400	polyclonal	-	rabbit	1.74	Wb, IF
Aviva Systems Biology	ARP51267_P050	QC17282–100430	AB_2160100	polyclonal	-	rabbit	0.50	Wb
Bio-Techne (R&D Systems)	MAB3995 *	CBLD0114061	AB_3658217	monoclonal	421015	rat	0.50	Wb
Bio-Techne (R&D Systems)	MAB39951 *	CJMT0123101	AB_11179085	monoclonal	925805R	mouse	0.50	Wb
Cell Signaling Technology	5933 **	2	AB_2886673	recombinant mono	D29E5	rabbit	0.13	Wb, IP, IF
GeneTex	GTX132552	42424	AB_2886682	polyclonal	-	rabbit	0.20	Wb
GeneTex	GTX132574	42508	AB_2888485	polyclonal	-	rabbit	0.22	Wb, IF
GeneTex	GTX634830 *	43346	AB_2888486	monoclonal	GT353	mouse	1.40	Wb
GeneTex	GTX634831 *	43346	AB_2809582	monoclonal	GT136	mouse	2.00	Wb, IF
Thermo Fisher Scientific	MA5–32297 **	AC4649685	AB_2802649	recombinant mono	SN07–21	rabbit	1.00	Wb, IF
Thermo Fisher Scientific	MA5–33099 *	AC4650511	AB_2849161	monoclonal	4H4	mouse	1.00	Wb, IF
Thermo Fisher Scientific	MA5–35258 **	AC4650228	AB_1310549	recombinant mono	1Q7F8	rabbit	0.06	Wb

Wb = western blot; IF = immunofluorescence; IP = immunoprecipitation,

* = monoclonal antibody,

** = recombinant antibody

**Table 3.** Table of secondary antibodies used.

Company	Secondary antibody	Catalog number	RRID (Antibody Registry)	Clonality	Concentration (μg/μL)	Working concentration (μg/mL)
Proteintech	HRP-Goat Anti-Rabbit Antibody (H + L)	RGAR001	AB_3073505	recombinant polyclonal	1.0	0.05
Proteintech	HRP-Goat Anti-Mouse Antibody (H + L)	RGAM001	AB_3068333	recombinant polyclonal	1.0	0.5
Thermo Fisher Scientific	HRP-Goat Anti-Rat Antibody (H + L)	31470	AB_228356	polyclonal	0.8	0.4
Cell Signaling Technology	Protein A, HRP conjugate	12291	NA	polyclonal	0.125	0.5
Proteintech	CoraLite Plus 555-Goat Anti-Rabbit Antibody (H + L)	RGAR003	AB_3073507	recombinant polyclonal	0.5	0.5
Proteintech	CoraLite Plus 555-Goat Anti-Mouse Antibody (H + L)	RGAM003	AB_3068539	recombinant polyclonal	0.5	0.5
Invitrogen	Alexa Fluor™ 555-Goat anti-Rat IgG (H + L)	A-21434	AB_141733	polyclonal	2	0.5

**Table 4.** Illustrations to assess antibody performance in all western blot, immunoprecipitation and immunofluorescence.

Western blot	Immunoprecipitation	Immunofluorescence
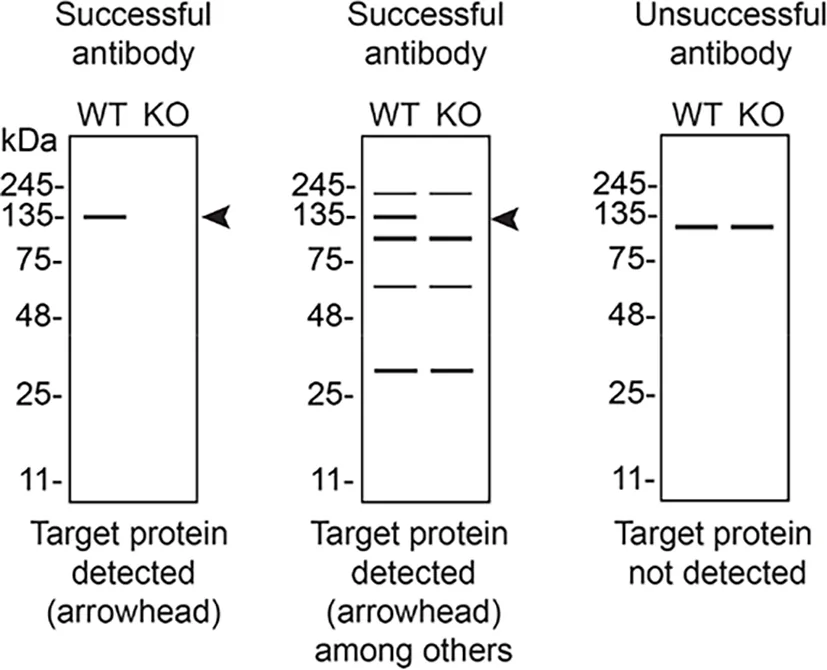	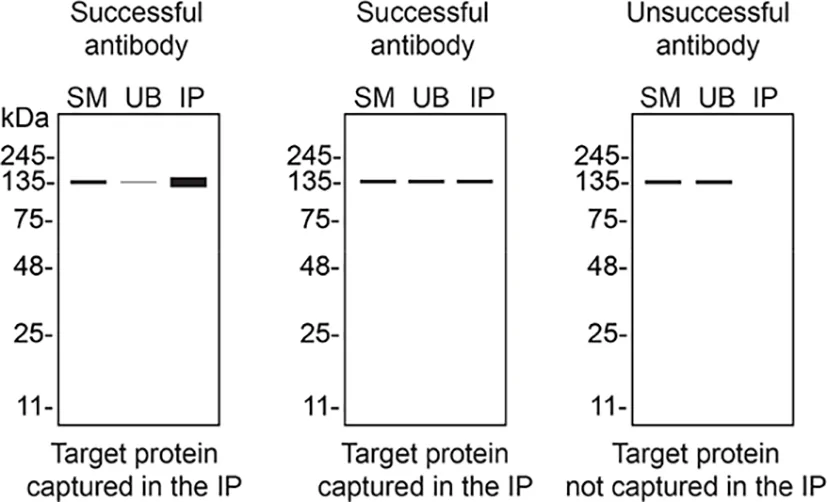	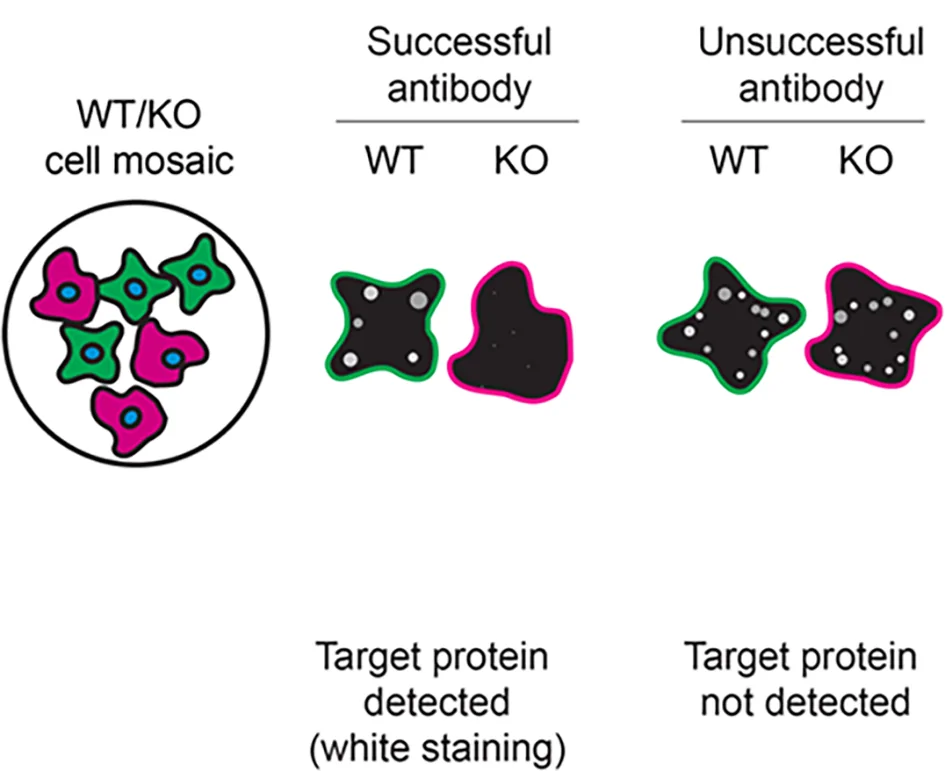

This table has been reproduced with permission from Ayoubi et al., Elife, 2023. ^8^

### Antibody screening by western blot

HEK-293T WT and *PARK7* KO cells were collected in RIPA buffer (25mM Tris-HCl pH 7.6, 150mM NaCl, 1% NP-40, 1% sodium deoxycholate, 0.1% SDS) (Thermo Fisher Scientific, cat. number 89901) supplemented with 1x protease inhibitor cocktail mix (MilliporeSigma, cat. number P8340). Lysates were sonicated briefly and incubated 30 min on ice. Lysates were spun at ~110,000 *x g* for 15 min at 4°C and equal protein aliquots of the supernatants were analyzed by SDS-PAGE and western blot. BLUelf prestained protein ladder (GeneDireX, cat. number PM008-0500) was used.

Western blots were performed with precast midi 10% Bis-Tris polyacrylamide gels (Thermo Fisher Scientific, cat. number WG1201BOX) ran with MES SDS buffer (Thermo Fisher Scientific, cat. number NP000202), loaded in LDS sample buffer (Thermo Fisher Scientific, cat. number NP0008) with 1x sample reducing agent (Thermo Fisher Scientific, cat. number NP0009) and transferred on nitrocellulose membranes. Proteins on the blots were visualized with Ponceau S staining (Thermo Fisher Scientific, cat. number BP103-10) which is scanned to show together with individual western blot. Blots were blocked with 5% milk for 1 hr, and antibodies were incubated O/N at 4°C with 5% milk in TBS with 0,1% Tween 20 (TBST) (Cell Signalling Technology, cat. number 9997). Following three washes with TBST, the peroxidase conjugated secondary antibody was incubated at a dilution of ~0.05 μg/ml in TBST with 5% milk for 1 hr at room temperature followed by three washes with TBST. Membranes were incubated with Pierce ECL (Thermo Fisher Scientific, cat. number 32106) prior to detection with the iBright™ CL1500 Imaging System (Thermo Fisher Scientific, cat. number A44240).

### Antibody screening by immunoprecipitation

Antibody-bead conjugates were prepared by adding 2 μg of antibody to 500 μl of Pierce IP Lysis Buffer from Thermo Fisher Scientific (cat. number 87788) in a microcentrifuge tube, together with 30 μl of Dynabeads protein A- (for rabbit antibodies) or protein G- (for mouse and rat antibodies) (Thermo Fisher Scientific, cat. number 10002D and 10004D, respectively). Tubes were rocked for ~1 h at 4°C followed by two washes to remove unbound antibodies.

HEK-293T WT were collected in Pierce IP buffer (25 mM Tris-HCl pH 7.4, 150 mM NaCl, 1 mM EDTA, 1% NP-40 and 5% glycerol) supplemented with protease inhibitor. Lysates were rocked 30 min at 4°C and spun at 110,000 *x g* for 15 min at 4°C. 0.5 ml aliquots at 2 mg/ml of lysate were incubated with an antibody-bead conjugate for ~1 h at 4°C. The unbound fractions were collected, and beads were subsequently washed three times with 1.0 ml of IP buffer and processed for SDS-PAGE and western blot on precast midi 10% Bis-Tris polyacrylamide gels.

### Antibody screening by immunofluorescence

HEK-293T WT and *PARK7* KO cells were labelled with a green and a far-red fluorescence dye, respectively (Thermo Fisher Scientific, cat. number C2925 and C34565). The nuclei were labelled with DAPI (Thermo Fisher Scientific, cat. Number D3571) fluorescent stain. WT and KO cells were plated on collagen-coated 96-well plate with optically clear flat-bottom (Perkin Elmer, cat. number 6055300) as a mosaic and incubated for 24 hrs in a cell culture incubator at 37oC, 5% CO _2_. Cells were fixed in 4% paraformaldehyde (PFA) (VWR, cat. number 100503-917) in phosphate buffered saline (PBS) (Wisent, cat. number 311-010-CL). Cells were permeabilized in PBS with 0,1% Triton X-100 (Thermo Fisher Scientific, cat. number BP151-500) for 10 min at room temperature and blocked with PBS with 5% BSA, 5% goat serum (Gibco, cat. number 16210-064) and 0.01% Triton X-100 for 30 min at room temperature. Cells were incubated with IF buffer (PBS, 5% BSA, 0,01% Triton X-100) containing the primary DJ-1 antibodies overnight at 4°C. Cells were then washed 3 × 10 min with IF buffer and incubated with corresponding Alexa Fluor 555-conjugated secondary antibodies in IF buffer at a dilution of 1.0 μg/ml for 1 hr at room temperature with DAPI. Cells were washed 3 × 10 min with IF buffer and once with PBS.

Images were acquired on an ImageXpress micro confocal high-content microscopy system (Molecular Devices), using a 20x NA 0.95 water immersion objective and scientific CMOS cameras, equipped with 395, 475, 555 and 635 nm solid state LED lights (lumencor Aura III light engine) and bandpass filters to excite DAPI, Cellmask Green, Alexa-555 and Cellmask Red, respectively. Images had pixel sizes of 0.68 x 0.68 microns, and a z-interval of 4 microns. For analysis and visualization, shading correction (shade only) was carried out for all images. Then, maximum intensity projections were generated using 3 z-slices. Segmentation was carried out separately on maximum intensity projections of Cellmask channels using CellPose 1.0, and masks were used to generate outlines and for intensity quantification. ^11^ Figures were assembled with Adobe Illustrator.

## Data Availability

Zenodo: Dataset for the DJ-1 antibody screening study ( 10.5281/zenodo.20161573). Data are available under the terms of the Creative Commons Attribution 4.0 International license (CC-BY 4.0).

## References

[1] Bao W Ge Y Huang J : DJ-1/PARK7 in Parkinson's disease: mechanisms of pathogenesis and therapeutic potential. *Neuroscience.* 2025;587:1–13. 10.1016/j.neuroscience.2025.09.025 40976433

[2] Junn E Jang WH Zhao X : Mitochondrial localization of DJ-1 leads to enhanced neuroprotection. *J. Neurosci. Res.* 2009;87(1):123–129. 10.1002/jnr.21831 18711745 PMC2752655

[3] Skou LD Johansen SK Okarmus J : Pathogenesis of DJ-1/PARK7-Mediated Parkinson's Disease. *Cells.* 2024;13(4). 10.3390/cells13040296 38391909 PMC10887164

[4] Ayoubi R Ryan J Gonzalez Bolivar S : A consensus platform for antibody characterization. *Nat. Protoc.* 2024;20:1509–1545. 10.1038/s41596-024-01095-8 39690206 PMC13054593

[5] Biddle MS Virk HS : YCharOS open antibody characterisation data: Lessons learned and progress made. *F1000Res.* 2023;12:1344. 37854875 10.12688/f1000research.141719.1 PMC10579855

[6] Laflamme C McKeever PM Kumar R : Implementation of an antibody characterization procedure and application to the major ALS/FTD disease gene C9ORF72. *elife.* 2019;8. 10.7554/eLife.48363 PMC6794092 31612854

[7] Alshafie W Fotouhi M Shlaifer I : Identification of highly specific antibodies for Serine/threonine-protein kinase TBK1 for use in immunoblot, immunoprecipitation and immunofluorescence. *F1000Res.* 2022;11:977. 10.12688/f1000research.124632.1 36415206 PMC9647147

[8] Ayoubi R Ryan J Biddle MS : Scaling of an antibody validation procedure enables quantification of antibody performance in major research applications. *elife.* 2023;12. 10.7554/eLife.91645 PMC10666931 37995198

[9] Bandrowski A Pairish M Eckmann P : The Antibody Registry: ten years of registering antibodies. *Nucleic Acids Res.* 2023;51(D1):D358–D367. 10.1093/nar/gkac927 36370112 PMC9825422

[10] Bairoch A : The Cellosaurus, a Cell-Line Knowledge Resource. *J. Biomol. Tech.* 2018;29(2):25–38. 10.7171/jbt.18-2902-002 29805321 PMC5945021

[11] Stringer C Wang T Michaelos M : Cellpose: a generalist algorithm for cellular segmentation. *Nat. Methods.* 2021;18(1):100–106. 10.1038/s41592-020-01018-x 33318659

