# Supplementary material for: A guide to selecting high-performing antibodies for DJ-1 ( PARK7) (Q99497) for use in western blot, immunoprecipitation, and immunofluorescence
Source: F1000Res. 2026 Jun 23;15:989. [Version 1] doi: 10.12688/f1000research.182633.1 (PMC13560570; doi:10.12688/f1000research.182633.1)
Supplement: Peer review history

# Reviewer report: Reviewer response for version 1

**DOI:** 10.5256/f1000research.201594.r497511

**Published:** 2026-08-27

**Authors:** Christian Tiede

**Affiliations:** 1 University of Leeds, Leeds, England, UK

This study compares 15 commercially available PARK7 antibodies (rather than the 16 indicated in the manuscript) for use in Western blotting, immunoprecipitation, and immunofluorescence assays using HEK293T wild-type and PARK7 knockout cells. The work provides a valuable resource for the community by systematically assessing antibody performance across multiple applications and highlighting substantial differences between reagents. In particular, Table 4 serves as a useful guide for selecting antibodies according to specific experimental requirements. Overall, the study is well conducted and, in my opinion, suitable for approval.

 I have one minor suggestion regarding the presentation of the immunoprecipitation data. Additional bands are visible in all IP blots, which likely correspond to the heavy and light chains of the antibodies used for the pulldown. While this is a common technical issue, it may make interpretation less straightforward for some readers. The authors may wish to consider using a secondary antibody specifically designed for immunoprecipitation applications, such as VeriBlot (Abcam, cat. no. ab131366), which does not detect denatured IgG heavy and light chains. Similar antibody characterization studies have successfully employed this approach, resulting in cleaner blots and facilitating data interpretation. Although this point does not detract from the overall quality of the study, addressing it could further improve the clarity of the presented results.

Are sufficient details of methods and materials provided to allow replication by others?

Yes

Is the rationale for creating the dataset(s) clearly described?

Yes

Are the datasets clearly presented in a useable and accessible format?

Yes

Are the protocols appropriate and is the work technically sound?

Yes

Reviewer Expertise:

Adhiron phage display, Adhiron protein production & characterisation.

I confirm that I have read this submission and believe that I have an appropriate level of expertise to confirm that it is of an acceptable scientific standard.

# Reviewer report: Reviewer response for version 1

**DOI:** 10.5256/f1000research.201594.r508763

**Published:** 2026-08-25

**Authors:** Deborah Moshinsky

**Affiliations:** 1 Institute for Protein Innovation, Boston, Massachusetts, USA

Mutations in the *PARK7* gene, which cause loss of function, are linked to early-onset familial Parkinson’s disease. Sixteen DJ-1 commercial antibodies were tested in western blot, immunoprecipitation, and immunofluorescence to determine whether the antibodies work under the specific protocols used in the study with the specific cell lines, including HEK-293T and isogenic knockouts. Performance of the antibodies varied within each application, and these data should be informative to researchers selecting antibodies to this target for use in their application. The authors give a reasonable explanation for why they do not engage in result interpretation. They are not experts in the biology of the targets but rather use this approach as a standardized way to provide additional antibody validation evidence for scientists to interpret themselves.

 Minor point - the authors fail to distinguish DJ-1 and PARK7, leaving the reader uncertain about the relationship.

 More generally - I completely understand the rationale for why the authors do not attempt to interpret the data, but the work would feel a little more substantial if some generalized conclusions are presented. For example, in this case, one could summarize the Western Blot data by saying that a number of antibodies yielded a band of approximately the expected size by Western blot; many with a single band and others with multiple bands.

Are sufficient details of methods and materials provided to allow replication by others?

Yes

Is the rationale for creating the dataset(s) clearly described?

Yes

Are the datasets clearly presented in a useable and accessible format?

Yes

Are the protocols appropriate and is the work technically sound?

Yes

Reviewer Expertise:

Antibody Characterization and Validation

I confirm that I have read this submission and believe that I have an appropriate level of expertise to confirm that it is of an acceptable scientific standard.

# Reviewer report: Reviewer response for version 1

**DOI:** 10.5256/f1000research.201594.r497512

**Published:** 2026-07-15

**Authors:** Jieya Shao

**Affiliations:** 1 Washington University in St Louis, St. Louis, USA

In this article the authors systematically compared the performance and specificity of fifteen commercially available anti-PARK7 antibodies using three standard experimental approaches (Western blot, immunoprecipitation, and immunofluorescence staining). This study is part of a large collaborative initiative to characterize commercial antibodies for different human proteins and offer unbiased guidance to researchers who can select the best antibodies for their specific experimental needs. As a Data Note, this paper successfully achieved its goal with carefully collected high-quality data, and the results are useful for the research community.

 1) Fifteen antibodies were characterized instead of the sixteen claimed.

 2) In the second paragraph of the Results section, the authors should change '...protein lysates were ran on SDS-PAGE...' to '...protein lysates were run on SDS-PAGE...' for grammatical accuracy.

 3) In Fig.2, nearly all IPs produced a protein band of approximately twice the size of monomeric PARK7. Although the authors are not supposed to interpret the results, this seems strange and could be an artifact. Also, what does the symbol “th antibody panel stand for?

Are sufficient details of methods and materials provided to allow replication by others?

Yes

Is the rationale for creating the dataset(s) clearly described?

Yes

Are the datasets clearly presented in a useable and accessible format?

Yes

Are the protocols appropriate and is the work technically sound?

Yes

Reviewer Expertise:

DNA replication, cancer biology, DNA damage response

I confirm that I have read this submission and believe that I have an appropriate level of expertise to confirm that it is of an acceptable scientific standard.
